# Lung Function and Organ Dysfunctions in 178 Patients Requiring Mechanical Ventilation During The 2009 Influenza A (H1N1) Pandemic

**DOI:** 10.1186/cc10369

**Published:** 2011-08-17

**Authors:** Fernando G Ríos, Elisa Estenssoro, Fernando Villarejo, Ricardo Valentini, Liliana Aguilar, Daniel Pezzola, Pascual Valdez, Miguel Blasco, Cristina Orlandi, Javier Alvarez, Fernando Saldarini, Alejandro Gómez, Pablo E Gómez, Martin Deheza, Alan Zazu, Mónica Quinteros, Ariel Chena, Javier Osatnik, Damian Violi, Maria Eugenia Gonzalez, Guillermo Chiappero

**Affiliations:** 1 Sociedad Argentina de Terapia Intensiva (SATI), The Registry of the Argentinian Society of Intensive Care, Niceto Vega 4617, (C1414BEA) Ciudad de Buenos Aires, Argentina; 2 Department Intensive Care, Clínica Olivos, Maipú 1660, (B1602ABQ), Vicente López, Buenos Aires, Argentina; 3 Department Intensive Care, CEMIC, Av. Las Heras 2900, (C1425AUM), Ciudad de Buenos Aires, Argentina; 4 Department of Adult Intensive Care, Hospital Nacional Alejandro Posadas, Marconi e Illia s/n, (B1706), El Palomar, Buenos Aires, Argentina; 5 Department Intensive Care, Hospital General de Agudos Velez Sarsfield, Calderón de la Barca 1550, (C1407AHH), Ciudad de Buenos Aires, Argentina; 6 Department Critical Care, Hospital Britanico, Perdriel 74, (C1280AEB) Ciudad de Buenos Aires, Argentina; 7 Department Intensive care, Hospital Lopez Lima, Gelonch 721, (R8332HLH) Gral. Roca, Río Negro, Argentina; 8 Department Critical care, Hospital Universitario Austral, Juan D. Perón 1500, (B1629ODT), Pilar, Buenos Aires, Argentina; 9 Department Intensive Care, Hospital General de Agudos "Donación Francisco Santojanni", Pilar 950, (C1408INH), Ciudad de Buenos Aires, Argentina; 10 Intensive Care Unit, Sanatorio de Los Arcos, Av. Juan B Justo 909, (C1425FSD), Ciudad de Buenos Aires, Argentina; 11 Critical Care Unit, Sanatorio Juncal, Av Almirante Brown 2779, (B1832) Temperley, Buenos Aires, Argentina; 12 Department Intensive Care, Hospital Bernardino Rivadavia, Av Las Heras 267, (C1425ASQ) Ciudad de Buenos Aires, Argentina; 13 Intensive Care Unit, Clínica de Especialidades, Corrientes 733, (X5901ACG), Villa María, Córdoba, Argentina; 14 Department Critical Care, Hospital General de Agudos, Juan A Fernández, Av Cervino 3356, (C1425AGP), Ciudad de Buenos Aires, Argentina; 15 Department Intensive Care, Hospital Lagomaggiore, Gordillo s/n, (5500), Mendoza, Argentina; 16 Department Intensive Care, Hospital Aleman, Av. Pueyrredón 1640, (C1118AAT), Ciudad de Buenos Aires, Argentina; 17 Department Intensive Care, Hospital Interzonal Guemes, Av. 2° Rivadavia 15.000, (B1404), Haedo, Buenos Aires, Argentina; 18 Department Intensive Care, Hospital Privado de la Comunidad, Córdoba 4545, (B7602CBM) Mar del Plata, Argentina; 19 Intensive Care Unit, Hospital Universidad Abierta Interamericana, Portela 2975, (C1069AAB), Ciudad de Buenos Aires, Argentina; 20 Intensive Care Unit, Sanatorio San Lucas, Belgrano 363, (B1642), San Isidro, Buenos Aires, Argentina; 21 Department Intensive Care, Hospital Interzonal General San Martin, Calle 1 n 1791, (B1900) La Plata, Buenos Aires, Argentina

## Abstract

**Introduction:**

Most cases of the 2009 influenza A (H1N1) infection are self-limited, but occasionally the disease evolves to a severe condition needing hospitalization. Here we describe the evolution of the respiratory compromise, ventilatory management and laboratory variables of patients with diffuse viral pneumonitis caused by pandemic 2009 influenza A (H1N1) admitted to the ICU.

**Method:**

This was a multicenter, prospective inception cohort study including adult patients with acute respiratory failure requiring mechanical ventilation (MV) admitted to 20 ICUs in Argentina between June and September of 2009 during the influenza A (H1N1) pandemic. In a standard case-report form, we collected epidemiological characteristics, results of real-time reverse-transcriptase--polymerase-chain-reaction viral diagnostic tests, oxygenation variables, acid-base status, respiratory mechanics, ventilation management and laboratory tests. Variables were recorded on ICU admission and at days 3, 7 and 10.

**Results:**

During the study period 178 patients with diffuse viral pneumonitis requiring MV were admitted. They were 44 ± 15 years of age, with Acute Physiology And Chronic Health Evaluation II (APACHE II) scores of 18 ± 7, and most frequent comorbidities were obesity (26%), previous respiratory disease (24%) and immunosuppression (16%). Non-invasive ventilation (NIV) was applied in 49 (28%) patients on admission, but 94% were later intubated.

Acute respiratory distress syndrome (ARDS) was present throughout the entire ICU stay in the whole group (mean PaO_2_**/**FIO_2 _170 ± 25). Tidal-volumes used were 7.8 to 8.1 ml/kg (ideal body weight), plateau pressures always remained < 30 cmH_2_O, without differences between survivors and non-survivors; and mean positive end-expiratory pressure (PEEP) levels used were between 8 to 12 cm H_2_O. Rescue therapies, like recruitment maneuvers (8 to 35%), prone positioning (12 to 24%) and tracheal gas insufflation (3%) were frequently applied. At all time points, pH, platelet count, lactate dehydrogenase assay (LDH) and Sequential Organ Failure Assessment (SOFA) differed significantly between survivors and non-survivors. Lack of recovery of platelet count and persistence of leukocytosis were characteristic of non-survivors. Mortality was high (46%); and length of MV was 10 (6 to 17) days.

**Conclusions:**

These patients had severe, hypoxemic respiratory failure compatible with ARDS that persisted over time, frequently requiring rescue therapies to support oxygenation. NIV use is not warranted, given its high failure rate. Death and evolution to prolonged mechanical ventilation were common outcomes. Persistence of thrombocytopenia, acidosis and leukocytosis, and high LDH levels found in non-survivors during the course of the disease might be novel prognostic findings.

## Introduction

On April 2009, a novel influenza A (H1N1) virus emerged in Mexico and spread rapidly across the world [1,2]. As of 17 June 2010, more than 214 countries had reported confirmed cases of infection with pandemic 2009 influenza A (H1N1) virus, including at least 18,156 deaths [3]. Unlike seasonal influenza, in which hospitalizations occur among patients younger than 2 and older than 65 years, or in those with underlying diseases [4], this novel virus affected otherwise healthy young and middle-aged adults and obese individuals [2,5]. Patients with previous respiratory disease, immunocompromised hosts and pregnant women were affected as frequently as with seasonal influenza [6-15]. Although a mild form of the disease was prevalent, it soon became evident that the 2009 influenza A (H1N1) virus could also provoke severe, acute respiratory failure requiring admission to the intensive care unit (ICU) for mechanical ventilation [16], which was reflected in the severe pathological injury found at autopsy [17].

The Argentinian population was greatly affected during the pandemic, with a total of 1,390,566 cases of influenza-like illness requiring 14,034 hospitalizations. Of the 11,746 confirmed cases of patients infected with the new strain, 617 died [18]. This represents a death rate per infection of 4.3% in hospitalized cases; an intermediate figure compared to 3.6% in Brazil, 1.2% in Chile, and approximately 6% in Uruguay, Colombia and Venezuela [19]. It should be noted that these numbers reflect great uncertainty, particularly with regard to case diagnosis. Lack of testing of mild disease and difficulties due to laboratory overload have also been well described [15,20]. These general problems have been acknowledged by experts [21].

The severity of disease was rapidly perceived by health authorities and scientific societies. Hence, a committee of experts of the Argentinian Society of Intensive Care Medicine decided to focus on the most acutely ill patients: those presenting with diffuse viral pneumonitis requiring mechanical ventilation. They designed an epidemiological study, recently-published, to determine risk factors and outcomes [15]; this is one of many series up to the present that have described epidemiological and clinical aspects of the 2009 influenza A (H1N1) pandemic [6-15].

There remains, however, a paucity of data published on physiological evolution during ICU stay [22]. This present study, concurrently planned with the first by the same committee of experts, thus aims to provide such information. Our objectives were: first, to characterize alterations of oxygenation, respiratory mechanics and the use of mechanical ventilation; second, to explore compliance with protective lung ventilation; and, finally, to assess the evolution of laboratory findings and organ dysfunctions throughout the course of the disease.

## Materials and methods

This was a multicenter, inception cohort study that included patients aged > 15 years admitted to the ICU with a previous history of influenza-like illness, evolving to acute respiratory failure that required mechanical ventilation during the 2009 winter in the Southern Hemisphere. These patients had confirmed or probable disease caused by the 2009 influenza A (H1N1) virus and were included in the Registry of Cases of the Argentinian Society of Intensive Care Medicine (SATI), created to characterize local aspects of the pandemic. On 27 June 2009, a form to collect online epidemiological data was posted on the official SATI website. A detailed description and analysis of this information was recently published [14].

There was also an optional, more comprehensive case-report form to complete, developed by experts of the SATI's Respiratory Committee for recording certain pre-specified variables throughout ICU stay, which included mechanical ventilation (MV), respiratory mechanics, oxygenation, blood chemistry and organ failure variables. This information was collected over 10 days and is analyzed in the present study.

Patients were characterized as confirmed, probable or possible cases of 2009 influenza A (H1N1) [20] according to the findings in the respiratory samples collected on admission. Some specimens, however, were not analyzed because laboratories soon became overloaded, especially at the beginning of the pandemic. As of 25 September 2009, the weekly update of the Ministry of Health reported that in patients ≥5 years with influenza-like illness, the 2009 influenza A (H1N1) virus had displaced other respiratory viruses in 93.4% of the samples processed [23,24]. As a result of this, probable and suspected cases were considered as caused by the novel virus and were so included in the study.

We collected dates of hospital and ICU admission, and of MV onset; demographics; risk factors for influenza A; actual weight; height; severity of illness (Acute Physiology And Chronic Health Evaluation II, APACHE II), organ failures (Sequential Organ Failure Assessment, SOFA); type of MV used, as noninvasive (NIV) and invasive; and date of intubation. Ideal body weight (IBW, ml/kg) and body mass index (BMI) were calculated; obesity was defined as a BMI > 30.

At MV onset (Day 0) and on Days 3, 7 and 10, until death or discharge, whichever occurred first, we recorded: (1) MV-related variables. (2) MV modes: volume-controlled ventilation (VCV); pressure-controlled ventilation (PCV); bilevel mode; pressure support ventilation (PSV); other. (3) Tidal volume (Vt, in ml/kg of IBW) (4) Pressures: peak, plateau pressures, total positive end-expiratory pressure (PEEP) and driving pressure (plateau pressure - PEEP), in cmH2O. (5) Static compliance (ml/cmH2O). (6) Respiratory rate (RR). (7) Inspired oxygen fraction (FIO2). (8) Use of adjuvants of MV: recruitment maneuvers, prone positioning, or tracheal gas insufflations. (9) Use of NIV: duration (hours); requirement of intubation; types of interfaces and of ventilators used (Bilevel/conventional). (10) Date of extubation; use of NIV for extubation failure, need of reintubation.(11) Date of tracheostomy 10. (12) Blood gases and acid-base variables. (13) PaO2/FIO2 relationship. (14) Lung infiltrates in CXR (in quadrants). (15) Use of oseltamivir, corticosteroids and neuromuscular blockers. (16) Blood chemistry. (17) Daily fluid balance. (18) SOFA score. (19) Cause of death.

The main outcome measure was hospital mortality; secondary outcomes were length of MV, of ICU (LOSICU) and of hospital (LOSHOSP) stays.

In case of missing observations, local study coordinators were contacted to provide the corresponding values. Proportions were calculated as percentages of existing data.

No assumptions for missing data were made.

### Statistical analysis

Statistical analysis was performed with SPSS 17.0 (SPSS Inc., Chicago, IL, USA). Data were analyzed for the entire population; for the subgroups of survivors vs. non-survivors; and for patients receiving NIV on admission vs. those who did not. Descriptive statistics used were: mean ± standard deviations (SD) and median and 25-75% interquartile ranges (IQR) for continuous data of normal and non-normal distribution, respectively; and percentages for categorical data. Differences between subgroups were analyzed with unpaired t test, Mann-Whitney U test, and Chi-square tests, as appropriate. A *P*-value of <.05 was considered statistically significant. A Kaplan-Meier curve was constructed to evaluate survival over the follow-up period.

Over time, normally distributed data were analyzed with two-way repeated measures of ANOVA. At the pre-specified time points, differences within the entire group and subgroups, and between subgroups, were tested using paired and unpaired t tests, respectively.

In non-normally distributed data, differences over time within the entire group and the subgroups were analyzed with Friedman's and Wilcoxon tests. Comparisons between subgroups at the pre-specified time points were tested with Mann-Whitney U test. The Bonferroni correction was used to adjustments for multiple comparisons.

The local Institutional Review Boards waived the need for informed consent, given the general lack of knowledge on the clinical and outcome characteristics of the ongoing pandemic and to the non-interventional study design.

## Results

### General characteristics (Table 1)

**Table 1:** Baseline characteristics at admission of the entire group, and comparisons between survivors and non-survivors.

	All (*n *= 178)	Survivors (*n *= 93; 52%)	Non-survivors (*n *= 85; 48%)	*P*-value
Age	44 ± 15	45 ± 16	43 ± 15	0.36
Male gender	98 (55%)	49 (53%)	49 (58%)	0.44
APACHE II	18 ± 7	17 ± 6	20 ± 7	0.001
SOFA	4 (5 to 8)	3 (5 to 7)	6 (4 to 8)	0.000
Days of symptoms	6.7 ± 4.3	5.8 ± 3	7.5 ± 5	0.014
BMI	28 ± 8	28 ± 6	29 ± 10	0.32
Obesity^1^	46 (26%)	17 (18%)	29 (34%)	0.016
COPD	28 (16%)	16 (17%)	12 (14%)	0.57
Asthma	10 (6%)	6 (6%)	4 (5%)	0.61
Other respiratory disease	5 (3%)	1 (1%)	4 (5%)	0.14
Chronic heart failure	10 (6%)	3 (3%)	7 (8%)	0.15
Other cardiac disease	8 (4%)	6 (6%)	2 (2%)	0.19
Ethilism/alcoholism	12 (7%)	8 (9%)	4 (5%)	0.30
Chronic hepatic disease	7 (4%)	1 (1%)	6 (7%)	0.04
Diabetes	16 (9%)	5 (5%)	11 (13%)	0.08
Chronic renal failure	13 (7%)	5 (5%)	8 (9%)	0.31
Immunosupression^2^	29 (16%)	10(11%)	19(22%)	0.04
Pregnancy	16 (9%)	7 (8%)	9 (11%)	0.47
Previous seasonal influenza vaccination	4 (4%)	2 (2%)	2 (2%)	0.93
Confirmed cases by RT-PCR testing	95 (53%)	49 (53%)	46 (54%)	0.85
Oseltamivir use^3^	174 (98%)	91 (98%)	82 (67%)	0.58
Corticosteroid use^4^	75 (42%)	40%	45%	0.51
Length of MV	10 (6 to 17)	11 (6 to 19)	10 (4 to 16)	0.05
LOS_ICU_	12 (7 to 19)	14 (10 to 22)	10 (4 to 16)	< 0.001
LOS_HOSPITAL_	16 (10 to 23)	21 (14 to 38)	11 (6 to 18)	< 0.001

BMI, body mass index; ICU, intensive care unit; LOS_ICU, _length of stay at the ICU, in days; LOS_HOSPITAL, _length of stay at the hospital, in days; MV, mechanical ventilation

^1 ^Obesity was defined as a BMI > 30

^2 ^Includes neoplasia, infection by human immunodeficiency virus, and autoimmune disease.

^3 ^For corresponding doses, see reference 14.

^4 ^Corticosteroids were prescribed in 36% of patients for septic shock as 300 mg/day of hydrocortisone. Ten percent received metilprednisolone for persistent ARDS; 3% for other reasons, and 2% were pregnant patients receiving antenatal dexametasone to accelerate maturation of fetal lungs.

Between 6 June and 28 August 2009, the SATI's online Registry included 337 patients admitted to 35 ICUs with confirmed/probable/possible diffuse viral pneumonitis caused by influenza A (H1N1), with acute respiratory failure requiring MV (14). Of these, 178 consecutive patients admitted to 20 ICUs were followed over time, and are presented in this study. To address any potential concern that unconfirmed cases could belong to a different population of patients, we performed a sensitivity analysis of clinical and outcome characteristics data after exclusion of these patients. The results of this analysis did not differ from those of the primary assessments, so the 178 patients are considered for evaluation.

**Figure 1:**
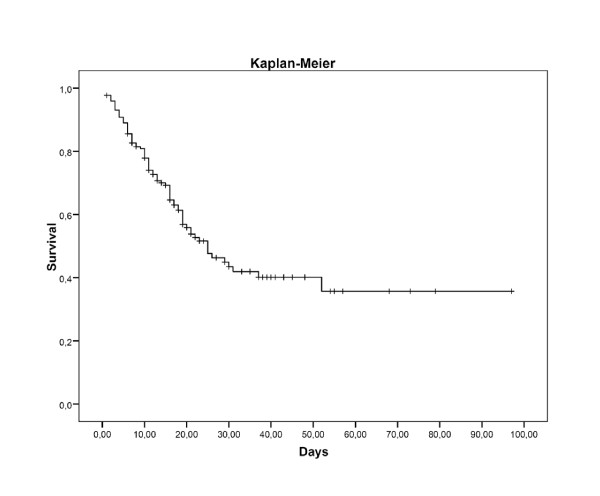
**Kaplan-Meier survival curve of the 178 patients with 2009 Influenza A (H1N1)**.

Briefly, patients were middle-aged, with no gender preponderance; they had a history of symptoms of nearly one-week duration and were ventilated at 1 [0 to -2] day after hospital admission. Pre-existent respiratory diseases, obesity, and diseases causing immunosuppression were the most frequent comorbid conditions; and prevalence of pregnancy was higher than in the general population, as expected [25]. Non-survivors were sicker on admission; duration of previous symptoms was longer; and organ failures were more severe. Obesity and immunosuppression were significantly more frequent as predisposing conditions. Ninety-three patients survived (52%) (See Figure 1).

### Clinical and mechanical ventilation-related variables (Table 2)

**Table 2:** Variables related to mechanical ventilation in all patients, and comparison between survivors and non-survivors.

	Day 0				Day 3				Day 7				Day 10			
	All	S	NonS	P	All	S	NonS	P	All	S	NonS	P	All	S	NonS	P
N	178	93	85		152	89	63		129	80	42		73	38	35	
Tidal volume (ml/kg of IBW)	7.8 ± 2	8.0 ± 2	7.7 ± 2	.35	7.9 ± 2	8.0 ± 2	7.8 ± 2	.46	7.9 ± 2	8.2 ± 2	7.5 ± 1	.02	8.1 ± 2	8.3 ± 2	7.9 ± 2	.35
Plateau pressure (cmH_2_O)	26 ± 7	25 ± 6	27 ± 8	.09	24 ± 7	24 ± 6	26 ± 8	.10	24 ± 7	23 ± 6	27 ± 8	.01	25 ± 8	24 ± 7	26 ± 8	.16
PEEP. (cm H_2_O)	10 (7 to 14)	10 (7 to 14)	10 (8 to 14)	.34	10 (7 to 14)	10 (7 to 12)	12 (7 to 14)	.05	10 (6 to 14)	8 ^a ^(5 to 12)	12 (8 to 15)	.003	10 (6 to 14)	8 ^b ^(6 to 12)	12 (8 to 15)	.008
Driving Pressure (cm H_2_O)	16 ± 6	15 ± 5	17 ± 6	_._.05	15 ± 6	14 ± 5	16 ± 6	.16	15 ± 6	14 ± 5	16 ± 7	.26	15 ± 7	14 ± 5	16 ± 8	.21
Respiratory rate (cycles/min)	21 ± 5	21 ± 5	21 ± 4	.66	21 ± 5	20 ± 4	22 ± 5	.22	21 ± 5	20 ± 4	22 ± 5	.15	20 ± 6	20 ± 5	23 ± 6	.03
PaO_2_/FIO_2_	136 (85 to 204)	146 (110 to 215)	125 (71 to 197)	.02	178 ^a ^(125 to 251)	220 ^a ^(156 to 287)	134 (84 to 185)	< 0.001	195 ^a ^(142 to 260)	230 ^a ^(172 to 292)	144 (88 to 190)	< 0.001	172 ^a ^(109 to 235)	212 ^a ^(164 to 270)	123 (80 to 160)	< 0.001
FIO_2 _(%)	80 (25 to 100)	70 (50 to 100)	100 (70 to 100)	< 0.001	60 ^a ^(40 to 70)	50 ^a ^(40 to 60)	65 ^a ^(50 to 100)	< 0.001	50 ^a ^(40 to 65)	50 ^a ^(40 to 55)	60^a ^(45 to 85)	< 0.001	50 ^a ^(41 to 70)	47 ^a ^(40 to 50)	80 ^a ^(50 to 100)	< 0.001

Data are presented as means ± standard deviations, and medians and (IQR)

Vt, Tidal volume. IBW, predicted body weight. S, Survivors. NonS, Non-survivors. P, Intergroup comparisons (between survivors vs. non-survivors) at each day are shown.

For intragroup comparisons over time: Days 3, 7 and 10 are compared to Day 0 values, in all patients, in survivors and in non-survivors.

^a ^*P *<.001; ^b ^*P *<.01

During the study period, the entire group had Vt values between 7.8 to 8.1 ml/kg of IBW, with plateau pressures remaining always < 30 cmH_2_O. Non-survivors displayed a trend towards lower Vt and higher plateau pressures, which differed significantly from survivors only at Day 7. Intermediate PEEP levels were used, and decreased in survivors from Day 3 onwards. Driving pressures were similar over time in all patients; only at admission did non-survivors exhibit higher values.

PaO_2_**/**FIO_2 _increased significantly over time in all patients and in survivors. It remained, however, < 200 in the whole group throughout the entire ICU stay due to non-survivor values. Non-survivors displayed significantly lower PaO_2_**/**FIO_2 _at all time points.

Lung infiltrates (in quadrants) peaked at day 3 (3.1 ± 1.0 vs. 2.9 ± 1 at Day 0, *P *< 0.01) and then decreased during the study in the entire group, especially at Day 10 (2.8 ± 1.1, *P *< 0.83 vs. Day 0), which reflected the improvement in survivors (3.1 ± 1.0 at Day 3 vs. 2.9 ± 1.0 at Day 10, *P *< 0.01).

**Figure 2:**
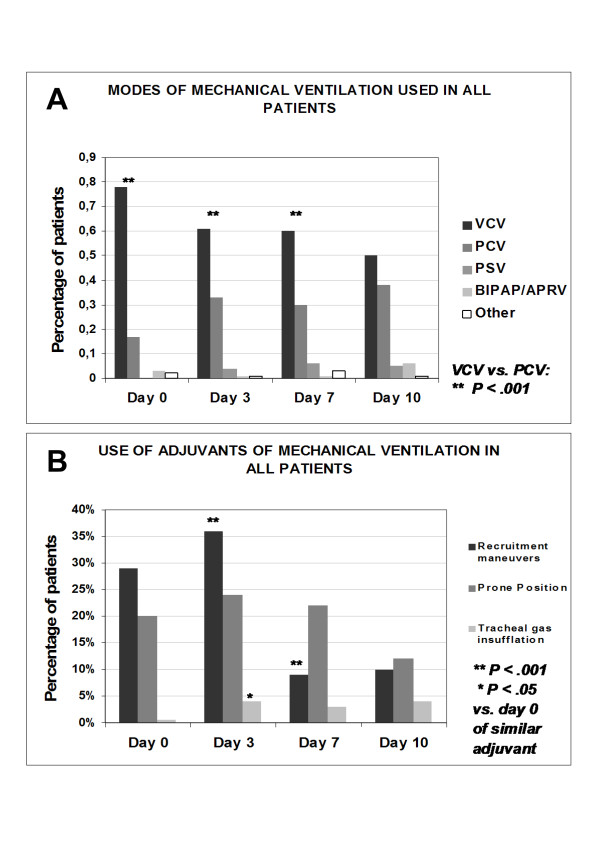
**Modes of mechanical ventilation (A)**. Use of adjuvants (B). All refer to the entire group.

In Figure 2, the utilization of ventilation modes and rescue therapies in the entire group are shown. Briefly, PCV use equaled VCV at Day 10, preceded by deterioration in oxygenation and respiratory mechanics: PaO_2_**/**FIO_2 _78 ± 24 vs. 128 ± 33, (*P *= 0.03); PaCO_2 _44 ± 4 vs. 35 ± 3 mmHg (*P *= 0.04); pH 7.29 ± 0.03 vs. 7.39 ± 0.05 (*P *= 0.05), and plateau pressures of 30 ± 2 vs. 25 ± 3 cmH_2_O (*P *= 0.03). Recruitment maneuvers became significantly more common in non-survivors at Day 3 (46%, vs. 29% in survivors; *P *= 0.03), as did prone positioning (24%, vs. 14%; *P *= 0.001). After that, only prone positioning remained significantly more used in non-survivors (at Day 7: 38%; vs. 14%, *P *= 0.004; and at Day 10: 25%; vs. 5%, *P *= 0.02). Six patients received tracheal gas insufflation; only one survived. Neuromuscular blockers were prescribed in 18% of patients on admission; and their use was subsequently more frequent in non-survivors (Day 3: 14% vs. 8%, *P *= 0.02; and Day 7: 14% vs. 8%, *P *= 0.04).

The main causes of death were refractory hypoxemia (64%); followed by multiorgan dysfunction syndrome (15%) and shock (10%). Prolonged mechanical ventilation and long ICU and hospital stays were frequent (Table 1). Tracheostomy was performed in 29 patients (16%) at Day 14 [11-21].

### Acid-base variables and fluid balance (Table 3)

**Table 3:** Oxygenation and acid-base variables, and fluid balance in all patients, and in survivors and non-survivors.

	Day 0				Day 3				Day 7				Day 10			
	All	S	NonS	P	All	S	NonS	P	All	S	NonS	P	All	S	NonS	P
N	178	93	85		152	89	63		129	80	42		73	38	35	
PaO_2 _(mmHg)	97 (71 to 124)	98 (76 to 120)	92 (67 to 129)	.50	94 (76 to 122)	98 (79 to 105)	87 (72 to 117)	.06	96 (79 to 115)	104 (84 to 125)	81 (73 to 102)	.001	96 (73 to 15)	106 (76 to 118)	84 (72 to 110)	.06
PaCO_2 _(mmHg)	44 ± 14	43 ± 12	46 ± 15	.22	44 ± 13	43 ± 13	46 ± 13	.14	47 ± 11	44 ± 10	51 ± 11	.02	47 ± 13	42 ± 10	52 ± 14	.001
Arterial pH	7.29 ± 0.1	7.31 ± 0.1	7.27 ± 0.1	.007	7.34 ± 0.1 ^a^	7.37 ± 0.1	7.29 ± 0.1 ^c^	.004	7.36 ± 0.1 ^a^	7.39 ± 0.1	7.33 ± 0.1	.002	7.37 ± 0.1 ^c^	7.41 ± 0.1	7.32 ± 0.1	.002
HCO_3 _(mEq/L)	21 ± 5	20 ± 6	22 ± 5	.16	24 ± 5 ^b^	24 ± 5 ^a^	22 ± 5	.02	27 ± 5 ^a^	27 ± 5	28 ± 5 ^b^	.55	27 ± 5 ^c^	27 ± 5	28 ± 7	.75
Fluid balance (ml/day)	785 (130; 1,802)	715 (98; 1,810)	1,000 (170; 1,800)	.99	1,050 (74; 210)	695 (-86; 2,025)	1,350 (505; 2,560)	.02	900 (-352;-1,785)	700 (-470;-1,770)	1,000 (-172; 1,810)	.75	129 ^b ^(-673; 1,100)	0 ^b ^(-800; 1,050)	757 (-450; 1,415)	.07

Data are presented as means ± standard deviations, and medians and (IQR)

NonS, Non-survivors. P, Intergroup comparisons (between survivors vs. non-survivors) at each day are shown. S, Survivors.

For intragroup comparisons over time: Days 3, 7 and 10 are compared to Day 0 values, in all patients, in survivors and in non-survivors.

^a ^*P *< .001; ^b ^*P *< .01; ^c ^*P *< .05

Arterial pH increased over time in the whole cohort and in both subgroups, perhaps secondary to general resuscitation measures. Despite this, non-survivors displayed significantly lower pH at all time points, owing to changes in base excess on Days 0 and 3, and to pCO_2 _elevations thereafter. Respiratory rates remained unchanged, only increasing at Day 10 in non-survivors; nevertheless, this corresponded to the highest pCO_2 _values, indicating the more severe respiratory compromise. Bicarbonate paralleled pH behavior.

Changes in fluid balance did not show clear trends: only at Day 10 they decreased significantly, expressing survivors' behavior.

### Use of noninvasive ventilation (Tables 4 and 5)

**Table 4:** Characteristics of patients receiving non-invasive ventilation on admission, and comparison to those receiving invasive ventilation

	NIV (*n *= 49)	Non-NIV (*n *= 129)	P
Proportion of the entire population	28%	72%	
Age (years)	40 ± 16	44 ± 15	0.26
Gender (male)	25 (51%)	73 (57%)	0.51
APACHE II	16 ± 6	19 ± 7	0.01
PaO_2_**/**FIO_2 _day0	129 (70 to 173)	141 (92 to 217)	0.12
PCO_2 _day0	42 (37 to 55)	42 (34 to 51)	0.23
COPD999	6/49 (12%)	22/129 (17%)	0.24
Asthma	2/49 (4%)	8/129 (6%)	0.58
CHF	1/49 (2%)	9/129 (7%)	0.20
Pregnancy	5/48 (10%)	11/128 (9%)	0.71
Immunosupression	3/49 (6%)	26/129 (20%)	0.03
Obesity^1^	12/49 (24%)	34/129 (26%)	0.80
Hospital mortality	19 (39%)	66 (51%)	0.14
Total duration of mechanical ventilation (days)^2^	12 (6 to 21)	10 (6 to 16)]	0.15
LOS_ICU_	15 (8 to 25)	11 (7 to 17)	0.06
LOS_HOSPITAL_	19 (11 to 25)	16 (8 to 23)	0.06

CHF, chronic heart failure; COPD, chronic obstructive pulmonary disease; LOS_ICU, _length of stay at the ICU, in days; LOS_HOSPITAL, _length of stay at the hospital, in days; NIV, non-invasive ventilation

^1 ^Obesity was defined as a BMI > 30

^2 ^Refers to the sum of the duration of NIV plus the duration of invasive ventilation.

**Table 5:** Variables associated with noninvasive ventilation success or failure.

	NIV success (*n *= 3)	NIV failure (*n *= 46)
Prior duration of symptoms (days)	3.6 ± 1.1	7.2 ± 3.8
Age	25 ± 10	42 ± 16
APACHE II	8 ± 3	17 ± 5
PaO_2_**/**FIO_2_	225 ± 115	140 ± 96
FIO_2_	0.35 ± 0.21	0.80 ± 0.22
Arterial pH	7.40 ± 0.03	7.31 ± 0.10
PaCO_2_	39 ± 2.5	46 ± 14
SOFA	2.6 ± 2	5.1 ± 2.7
LOS_ICU_	13 (4 to 22)	16 (8 to 23)
LOS_HOSPITAL_	16 (4 to 27)	19 (12 to 32)

LOS_ICU, _Length of stay at the ICU, in days; LOS_HOSPITAL, _Length of stay at the hospital, in days; NIV, non-invasive ventilation. Due to the small number of patients that did not require intubation and mechanical ventilation (*n *= 3), statistical analysis was not performed.

Forty-nine patients (28%) underwent a trial of NIV on admission; they were significantly less ill and had a lower incidence of immunosuppression. Oxygenation and outcome variables were similar to those of patients not receiving NIV.

Sixty-one percent of patients (*n *= 30) receiving NIV survived; duration of NIV was of 8 (2 to 18) hours. There were no differences between survivors and non-survivors in the duration of the procedure, or in the type of interface or respirator used. Of note, most patients on NIV (46 out of 49; 94%) had to be intubated and ventilated invasively for hypoxemic failure. Characteristics associated to NIV success/failure are shown in Table 5.

NIV was also used for treating post-extubation respiratory failure in 12 of 178 patients (7%), with success (reintubation not needed) in 8 cases (66%).

### Blood chemistry and organ failures

The most consistent changes over time were found in platelet count, which increased significantly in the whole cohort (*P *< 0.000 for Days 3, 7 and 10 vs. Day 0), secondary to elevations in survivors. At all time points, platelets differed between survivors and non-survivors. Conversely, white blood cell count showed a progressive increase in the whole group (*P *< 0.000 for Days 7 and 10 vs. Day 0), due to elevations in non-survivors.

Creatine-kinase and markers of liver injury (alanine/aspartate aminotransferases, serum bilirubin; not shown) were mildly elevated and displayed no substantial changes. On the contrary, lactate-dehydrogenase levels were significantly higher in non-survivors throughout the study. Creatinine levels were stable over the period, but were significantly higher in non-survivors on Days 0 and 3. Finally, SOFA score diminished over time in all patients (*P *< 0.000 for Days 7 and 10 vs. Day 0), as a result of the decrease in survivors. SOFA was significantly lower in survivors throughout the study.

**Figure 3:**
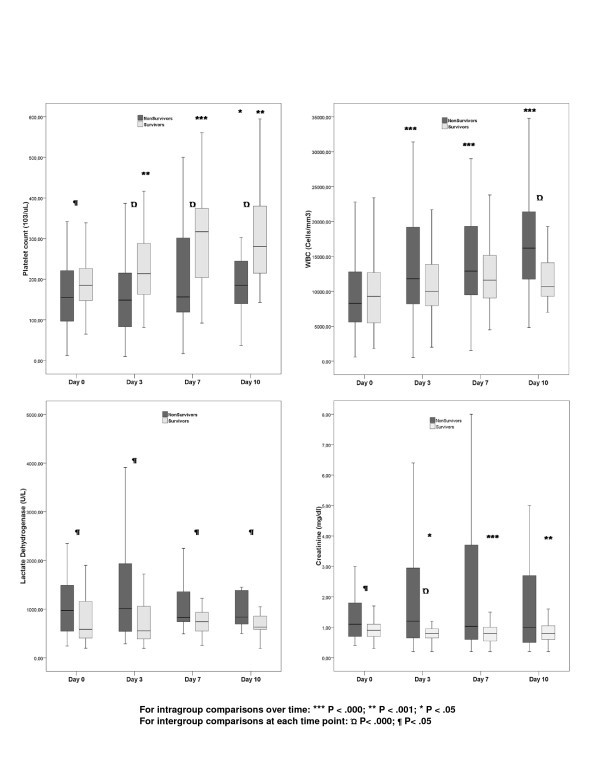
**Temporal patterns of platelet and white blood cell counts, lactic-dehydrogenase and serum creatinine**.

In Figure 3, the differences between survivors and non-survivors are displayed.

## Discussion

We report on a large, prospective cohort of 2009 influenza A (H1N1) patients that were mechanically ventilated for acute respiratory failure due to diffuse pneumonitis during the pandemic in Argentina. Though most were middle-aged, previously healthy adults, patients with preexistent lung disease, immunosuppression, obesity and pregnancy were also affected. Mortality was high and evolution to chronic critical illness was common, as shown by prolonged mechanical ventilation, high needs of tracheostomy, and lengthened ICU and hospital stays.

Patients had characteristically a history of protracted symptoms and displayed severe compromise of oxygenation compatible with ARDS throughout the study period, which only improved in survivors. At all time points, PaO_2_**/**FIO_2 _differed significantly between survivors and non-survivors, requiring higher FIO_2 _and PEEP in this last subgroup. Yet the levels of applied PEEP were only in the intermediate range, similar to mean values of 8.7 cmH_2_O of PEEP in an international study on mechanical ventilation [26], which may explain the relatively high FIO_2 _used in our study. Driving pressures were similar in both subgroups most of the time, suggesting an intention to limit alveolar excursion as part of a protective strategy.

It is striking that, as has been described in similar studies on mechanical ventilation performed during the 2009 influenza A (H1N1) pandemic [6,7], tidal volumes used were between 7.5 and 8.3 ml/kg IBW, certainly higher than the 6 ml/kg demonstrated as being lung-protective [27]. Indeed, barriers to implementing low-tidal volume have been identified and might explain physician behavior [28]. Despite this, plateau pressures did remain below 30 cmH_2_O [29], indicating that lung compliance might have been preserved. Perhaps clinicians focused on plateau pressures rather than on tidal volumes [30] since it still remains unclear which should be limited to avoid ventilator-induced lung injury [31]. We, like others [6,7,32,33], could not find differences in utilized tidal volumes between survivors and non-survivors. Even so, non-survivors tended to display lower values, probably reflecting physician efforts to intensify protective ventilation strategies in the most severely compromised. Some researchers [34,35] have suggested that allowing higher tidal volumes in a population of young and previously healthy patients with strong ventilatory drive might reveal an attempt to restrain heavy sedation and neuromuscular blocker use. Notwithstanding this, we believe that these findings may also represent clinicians' inadequate prescription, as described in other scenarios [36].

Not unexpectedly, VCV was the most common ventilator mode used. PCV use increased throughout the study period, peaking at Day 10. This is in contrast with the recently identified trend towards decreased PCV utilization. Transition to PCV mode was associated with preceding physiological worsening, so clinicians might have perceived PCV utilization as part of a global lung-protective strategy [37].

Refractory hypoxemia was the main cause of death. As in other studies [6,7,11], rescue therapies were frequently applied, with utilization highest 72 hours after admission. Recruitment maneuvers and prone positioning were the primary adjuvants utilized; ECMO and HFOV are currently not available in Argentina. A prolonged mechanical ventilation course was frequent as reported elsewhere [6].

NIV was the first ventilation approach in 28% of cases, with 94% later requiring invasive ventilation, as has been documented in other studies [6,7,11]. These common experiences should caution against delaying proper ventilatory support in this group, given that rapid deterioration is common. A recent meta-analysis suggests that NIV does not decrease the need for intubation, so evidence to support its use in severe ARDS is questionable [38]. In our study, improved outcomes with NIV could be due to milder disease, evidenced by APACHE II. The small number of patients that were not intubated precludes a statistical analysis; however, they were younger, with less severe disease and better oxygenation.

Significant changes in fluid balance were late and reflected changes in survivors. Negative fluid balances could never be obtained, perhaps suggesting a continuing need for hemodynamic support: 72% of patients presented with shock [14]. On the whole, fluid balances remained between those achieved by "liberal" and "conservative" strategies of the fluids and catheters treatment trial, depending on the day evaluated [39]. Thus far, it is not clear whether the negative fluid balance has a causal role in improving outcome in ALI/ARDS, or if it simply expresses the global recovery of patients.

Another important finding was that arterial pH consistently and significantly differed between survivors and non-survivors, as described elsewhere [40,41]. During the first 72 hours acidosis had a major metabolic component, likely as a sign of hemodynamic impairment. After the first week, respiratory acidosis ensued, indicating either the effects of protective ventilation, or merely deterioration due to progressive shunt, profound ventilation/perfusion mismatch and increased deadspace.

With respect to blood chemistry, the usual findings of thrombocytopenia, leukocytosis and mildly elevated creatine-kinase blood levels were present [21,42]. Regrettably, the lymphocyte count was not recorded. In viral infections, thrombocytopenia occurred frequently. Although the mechanisms by which the 2009 influenza A (H1N1) virus causes thrombocytopenia are unknown, its lack of resolution is a marker of poor prognosis. Both leukocytosis and leucopenia have been found in hospitalized patients with 2009 influenza A (H1N1) [2,43]; in our study, persistent leukocytosis was associated with increased mortality. LDH elevations have been previously described in fatal cases [2], which corresponded to our finding of higher LDH levels in non-survivors at all time points. Such elevations have also been reported in seasonal influenza [44]. In experimental studies, increased LDH is a marker of human fetal membrane cell apoptosis induced by influenza virus [45]. Finally, multiorgan failure was frequent, and predictably more severe in non-survivors.

This study has several strengths: first, the clinical characteristics and time course of pandemic 2009 influenza A (H1N1) are thoroughly described and analyzed. Second, data were collected prospectively in consecutive patients and with a standardized case-reporting form, representing a large, nationwide cohort. Third, temporal patterns of mechanical ventilation use, acid-base and blood chemistry variables, as well as fluid balance and organ failures, are carefully analyzed. Prognostic implications are highlighted. Finally, we present the largest experience with NIV use during the pandemic.

Study limitations include the focus on mechanically ventilated patients, excluding less severe cases also admitted to the ICU. Many cases could not be confirmed because laboratories were overwhelmed with clinical samples, which is also described elsewhere [7,14]. Data about transmission to healthcare workers were not recorded, especially regarding NIV. Currently, most information about its use during an epidemic relies upon expert opinion [46].

## Conclusions

In 178 patients with diffuse viral pneumonitis caused by the 2009 influenza A (H1N1) virus admitted to the ICU and followed over time, ARDS was the rule, requiring high ventilation support and frequent use of rescue therapies. Death, organ failures, and evolution to prolonged mechanical ventilation were common. In most cases, noninvasive ventilation failed to prevent endotracheal intubation. Finally, elevated LDH levels, lack of recovery of platelet count and persistent acidosis and leukocytosis in non-survivors behaved as prognostic findings.

## Key messages

• In 2009 influenza A (H1N1) patients, hospital admission with prompt indication of mechanical ventilation - a marker of severe disease - was associated with a history of symptoms of nearly one-week duration.

• An initial NIV trial was not effective to avoid intubation in most patients; thus, this ventilation approach should likely be discarded in this setting.

• Mortality and morbidity were frequent: death was common and was mainly caused by persistent, refractory hypoxemia. Prolonged mechanical ventilation and ICU and hospital stays were typical.

• pH, platelet count, LDH and SOFA differed significantly between survivors and non-survivors over time. Lack of recovery of platelet count and persistence of leukocytosis might be markers of poor prognosis.

• Every effort should be done to increase adherence to protective ventilation in the real world.

## Abbreviations

ALI: acute lung injury; ARDS: acute respiratory distress syndrome; BMI: body mass index; CXR: plain chest X-ray film; IBW: ideal body weight; ICU: Intensive Care Unit; LDH: lactate dehydrogenase assay; LOS: length of stay; MV: mechanical ventilation; NIV: non-invasive ventilation; PaO2/FIO2: relation between patient arterial pO_2 _and inspired oxygen fraction used; PCV: pressure-controlled ventilation; PEEP: positive end-expiratory pressure; PSV: pressure support ventilation; RR: respiratory rate; RT-PCR: real-time reverse-transcriptase--polymerase-chain-reaction; SATI: Argentinian Society of Intensive Care; SOFA: Sequential Organ Failure Assessment; VCV: volume-controlled ventilation; Vt: tidal volume.

## Competing interests

The authors declare that they have no competing interests.

## Authors' contributions

FGR conceived and coordinated the study. FGR and EE drafted the manuscript and performed the statistical analysis. FGR, FV, RV, AG, DV and GC participated in the design of the study. FGR, EE, FV, RV, LA, DP, PV, MB, CO, JA, FS, AG, PEG, MD, AZ, MQ, AC, JO, DV, MEG, GC and the rest of the members of the Registry performed the acquisition of data. All authors read and approved the final manuscript.
